# Reading Comprehension in Older Adults—Effects of Age, Educational Level, and Reading Habits

**DOI:** 10.3390/jintelligence13010004

**Published:** 2024-12-31

**Authors:** Bernardo Riffo, Carlos Rojas, Andrea Helo, Mónica Véliz, Paula Urzúa, Gloria Gutierrez, Ernesto Guerra

**Affiliations:** 1 Department of Spanish, Universidad de Concepción, Concepción 4030000, Chile; bernardo@udec.cl (B.R.); mveliz@udec.cl (M.V.); glorigutierrez@udec.cl (G.G.); 2 Department of Health Rehabilitation Sciences, Universidad del Bío-Bío, Chillán 3780000, Chile; 3 Department of Speech and Hearing Science, Universidad de Chile, Santiago 8320000, Chile; ahelo@uchile.cl; 4 Center for Advanced Research in Education, Institute of Education (IE), Universidad de Chile, Santiago 8320000, Chile; ernesto.guerra@ciae.uchile.cl; 5 Department of Neurosciences, Universidad de Chile, Santiago 8320000, Chile; 6 School of Speech and Hearing Science, Universidad de Las Américas, Concepción 4030000, Chile; paula.urzua.carmona@edu.udla.cl

**Keywords:** older adults, reading comprehension, fourth-age, age, educational level, reading habits

## Abstract

Older adults in the third-age group (60–79 years) maintain reading comprehension skills similar to those of younger adults, but little is known about individuals in the fourth age (80+ years). This study investigates differences in reading comprehension in a between-group design. We evaluated a sample of 150 older adults, comprising 86 third-age and 64 fourth-age participants. We examined the influence of sex, cognitive functioning, formal education, self-perceived reading difficulties, and reading habits on their text comprehension abilities. The results show that fourth-age adults have a significant decline in reading comprehension compared to third-age adults. Strong reading habits were positively associated with better comprehension across both groups, suggesting that regular reading may buffer against age-related cognitive decline. Poor readers read less frequently and perceived greater difficulty with the tasks. Cognitive functioning and education did not significantly influence comprehension—possibly due to the generally low education levels in the sample. However, strong reading habits appeared to compensate for these limitations. These findings suggest a potential protective role of lifelong reading habits and highlight the need for interventions to support reading skills in older adults, especially those with lower educational backgrounds. Future research should explore these dynamics further to enhance cognitive resilience in the oldest populations.

## 1. Introduction

Reading comprehensively is an essential skill for members of literate societies. In numerous situations of daily life, citizens must read texts of various genres in different contexts and for diverse purposes. Older adults are no exception; on the contrary, evidence suggests that reading is necessary for the successful performance of daily activities and contributes to the autonomy of older people (Chang et al. 2021).

During reading, several sub-processes can be distinguished. Kintsch’s psycholinguistic model (Kintsch 1998; Kintsch and Rawson 2005) explains text comprehension as a multi-layered process. At the surface representation level, readers process the text literally, focusing on the syntactic structure and lexical features of the words and sentences as they appear. Subsequently, they construct a “textbase”, extracting the fundamental propositions and explicit semantic relationships that form the basic content of the text. Then, they integrate this information into a broader “situation model” through both local sentence-level and global thematic coherence. Therefore, older readers, as well as the general population, require various cognitive skills to perform these multiple tasks that occur almost simultaneously. This involves several cognitive mechanisms, such as visual perception, memory, and executive functions, as well as different levels of language processing. Readers must draw on their cognitive and linguistic skills, as well as on knowledge accumulated through experience.

Recent research has further examined how cognitive and linguistic reserves influence reading comprehension in adults. Freed et al. (2017) investigated variables such as word decoding, working memory capacity, general reasoning, verbal fluency, perceptual speed, inhibition, and language experience. Their findings revealed that general reasoning and language experience were the only variables with direct effects on reading comprehension, emphasizing the significant role of accumulated linguistic experience and vocabulary. Building on this work, Goring et al. (2021) reanalyzed these data and identified overlapping relationships among several of these variables. They proposed that reading comprehension is inherently complex, involving dynamic interactions between different cognitive abilities, thereby challenging the notion of the independency of these variables. Together, these findings highlight the importance of considering both cognitive abilities and linguistic reserves, such as crystallized intelligence, when studying reading comprehension.

The existing literature shows that aging is associated with changes characterized by a general slowing and a decline in cognitive abilities, particularly in solving novel problems, reasoning abstractly, and processing information quickly—commonly referred to as fluid intelligence (Salthouse 1996; Lindenberger 2014; Schubert et al. 2020). These changes are widely attributed to the natural decline of the nervous system, which has significant implications for the functioning of sensory–perceptual mechanisms (Baltes and Lindenberger 1997) and the cognitive system, largely due to deficits in neural transmission (Burke et al. 2002; Mirman and Britt 2014). Cognitively, older adults experience declines in attention, memory, and executive functions such as working memory (Just and Carpenter 1992; Klencklen et al. 2017) and inhibitory control (Hasher et al. 1991; Rey-Mermet and Gade 2018; Verhaeghen and Cerella 2002). Linguistically, older adults show a decline in speech perception (Tun et al. 2009) and lexical production (Rojas et al. 2023a, 2023b) and difficulties in complex sentence processing (Stewart and Wingfield 2009).

However, older adults also see an increase in their reserves of experience, knowledge, and passive vocabulary, commonly known as crystallized intelligence, which refers to the accumulated knowledge and skills gained through education and experience, allowing them to maintain or even improve cognitive performance to some extent (Cooley 2020). Specifically, they show an increase in passive vocabulary, adequate discourse comprehension at the macrostructural level (Radvansky et al. 2001; Radvansky et al. 2003), and preserved semantic abilities (Burke and Shafto 2008; Chin et al. 2015; Artuso and Belacchi 2021). Thus, the situation presents an asymmetric configuration, with some aspects showing an undeniable deterioration, while others seem to be well preserved or even improved (Burke and Shafto 2008). This asymmetry may help to explain the linguistic–communicative functionality observed in cognitive healthy older adults, as they usually appear to efficiently compensate for the fluid intelligence losses using their crystallized intelligence reserves through various self-regulatory strategies (Chin et al. 2015; Stine-Morrow et al. 2016).

Thus, the functional (and structural) changes that affect various cognitive processes during aging (Duncan et al. 2017; Margrett et al. 2016; Mitchell et al. 2013) define old age as a distinct stage of human development. However, among older adults, there appear to be two very marked groups in terms of their cognitive capabilities: the third age (60–79 years) and the fourth age (from 80 years) (Höpflinger 2017; Higgs and Gilleard 2017, 2022). In the latter phase, the fourth age, typical aging changes deepen, giving rise to a more pronounced linguistic and cognitive performance decline, as well as signs of more advanced deterioration (Margrett et al. 2016). At the same time, this group is characterized by a high heterogeneity background; as they have lived longer, people above 80 years have a larger collection of experiences than third-age adults. As a consequence, the cognitive performance of this group shows a high dispersion, with very strong individual differences (Margrett et al. 2016). Because this is a new demographic phenomenon in human history (never have there been so many people of such advanced age; Higgs and Gilleard 2017, 2021, 2022), our knowledge of the developmental trajectory of this phase of the life cycle is still very limited.

In this context, investigating reading comprehension at a very late stage of the life cycle (fourth age) provides a unique opportunity to observe processes that do not occur at any other time in life. Reading comprehension research in people above 80 years of age is scarce; thus, this investigation is needed to identify how these the cognitive changes above described might influence this process during the fourth age.

### 1.1. Reading Comprehension Among Older Adults

While research on reading comprehension in the fourth age is nearly non-existent (but see Norman et al. 1992), there is some evidence regarding this skill in aging populations younger than 80 years (e.g., Burke and Shafto 2008; Gordon et al. 2016; Martín-Aragoneses et al. 2023). Studies on reading comprehension suggest that despite some memory deficits, comprehension remains well-preserved in the third-age group. Compared to younger individuals, older adults in this age group demonstrate comparable performance levels (Alexander et al. 2012; Zelinski and Kennison 2007). Additionally, several studies show that people between their 60s and 70s exhibit similar effects as young adults, particularly in terms of word frequency and predictability (Kliegl et al. 2004; Laubrock et al. 2006; Rayner et al. 2006). However, a closer look reveals notable differences between young adults and older individuals (see Gordon et al. 2016), including slower processing at the local level (sentence) and overall performance (paragraph or entire text) in subsequent recall (Miller and Stine-Morrow 1998; Smiler et al. 2003; Payne and Stine-Morrow 2012, 2014). Likewise, Radvansky et al. (2003) found that older adults demonstrated weaker memory for surface details and text-based knowledge (the explicit content of the text) compared to younger adults, while older adults exhibited equal or superior memory for the situation model (the broader meaning or message of the text). They suggested that older adults can temporarily construct sufficient surface and text-based representations (explicit meanings) as scaffolding for building a situation model (the overall reference of the text); once the goal is achieved, this scaffolding fades, freeing up memory resources (Radvansky et al. 2001). Based on these and other findings, the self-regulated model of language processing proposes that third-age adults compensate for age-related deficits—primarily affecting fluid intelligence—by utilizing crystallized intelligence reserves through strategic resource allocation, particularly in the form of extended processing time as a reflection of attention (Stine-Morrow et al. 2006).

What about reading comprehension performance during the fourth age? As mentioned earlier, most cognitive deficits that support the reading process tend to deepen in the fourth age, leading to more pronounced declines in both cognitive and linguistic performance. However, the decline in this population shows greater variability, likely due to cognitive reserves influenced by genetic factors (Laukka et al. 2013) and environmental factors, such as lifestyle habits like reading (Bavishi et al. 2016; Ng et al. 2019; Rothbauer and Dalmer 2018; Rodrigues et al. 2021). Despite this, research on this later stage of life remains limited. This raises the question: Are the effects of aging in the fourth age significant enough to differentiate it from the third age in terms of reading comprehension performance?

### 1.2. Role of Education Level and Reading Habits

Regarding older adults, there is considerable evidence for the maintenance of verbal performance in the cognitively healthy, which has been likened to crystallized intelligence (Margrett et al. 2016; Mitchell et al. 2013; Rojas et al. 2023a). However, in addition to the role of the crystallized intelligence as a cognitive reserve (Cooley 2020), several environmental factors are associated with the maintenance of verbal abilities in old age, including level of formal education, income, reading habits, and the type of work and intellectual activity that people have developed throughout their lives (Bavishi et al. 2016; Ng et al. 2019; Rothbauer and Dalmer 2018; Rodrigues et al. 2021).

Educational level, for example, is related to older adults’ reading comprehension in several ways (Malcorra et al. 2022b; Kavé et al. 2012; Sörman et al. 2018). First, higher levels of education are associated with stronger decoding skills, enabling older adults to read and process words and sentences faster and more accurately, which contributes to a more effective reading comprehension process (Kavé et al. 2012; Welcome and Meza 2019; Mokri et al. 2013). Second, there is evidence that older people with higher levels of education are likely to have larger vocabularies and more extensive knowledge in different areas (Ng et al. 2019). This allows them to comprehend complex texts more easily because they are familiar with a wider range of terms and concepts. Older adults with higher levels of education also tend to have a better ability to infer the meaning of unfamiliar words from context and to understand the structure and organization of texts. This helps them extract meanings and make connections between the ideas presented in the text. In addition, they demonstrate a greater ability to analyse and synthesize the information they encounter in texts, such as identifying main ideas, extracting relevant details and relating concepts to one another (Malcorra et al. 2022a; Sörman et al. 2018). Finally, people with higher levels of education often develop stronger reading habits and are more motivated to read regularly (Ng et al. 2019; Sörman et al. 2018). This leads to more practice and experience in reading comprehension, which in turn improves their reading comprehension skills (Kavé et al. 2012; Welcome and Meza 2019; Mokri et al. 2013).

Reading habits represent a particular type of intellectual activity whose benefits are far-reaching. Particularly, an important relationship has been found between reading habits throughout life (Malcorra et al. 2022a; Sörman et al. 2018) and performance in reading comprehension of older adults (Payne et al. 2012). Older people’s reading habits can have several important effects on reading comprehension, including improving reading fluency, which allow them to process and comprehend text more efficiently. Reading regularly exposes older people to a variety of new words and terms, which helps to expand their vocabulary, so that a larger vocabulary facilitates comprehension of more complex texts and improves the ability to make inferences (Perfetti and Stafura 2014; Guerra and Kronmüller 2020), as well as accessing the meaning of unfamiliar words from context (Bann et al. 2006; Chang et al. 2021; McLoughlin 2021). Moreover, evidence suggests that the habit of reading can contribute to the maintenance and improvement of cognitive skills such as memory, attention, and critical thinking in older adults, which are fundamental tools for effective reading comprehension (Bavishi et al. 2016). By being exposed to a wider variety of writing styles and text genres, they develop skills in identifying themes, analyzing characters, and understanding the structure of texts, which improves their overall comprehension (Bann et al. 2006; Payne et al. 2012; Sörman et al. 2018; Chang et al. 2021; McLoughlin 2021). Interestingly, people who read regularly and have done so for many years are not only better readers and perform better on reading comprehension tests; they also live longer and better lives (Bavishi et al. 2016).

Interestingly, research on both school and university students indicates that women generally outperform men in reading (Spencer and Cutting 2021; Ngongare et al. 2020) and are perceived as having stronger reading habits (Espinoza and Strasser 2020; Jabbar and Warraich 2023). This pattern seems to persist into older adulthood, with evidence showing that older women continue to read more frequently and maintain better reading habits compared to their male counterparts (Sörman et al. 2018). Therefore, while the primary focus of this study is on reading comprehension in individuals of advanced age, we will also examine how sex differences in reading abilities and habits emerge across different age groups.

In sum, reading comprehension in older adults is shaped by a complex interplay of cognitive factors, including the decline of fluid intelligence and the compensation through crystallized intelligence, along with external factors such as educational level and lifelong reading habits. Additionally, reading habits might be influenced by sex. While reading comprehension is relatively well-preserved in the third age (60–79 years), significant challenges arise in the fourth age (80+ years), where the variability in cognitive and linguistic performance becomes more pronounced. Nevertheless, higher education levels and consistent reading practices appear to buffer some of the age-related declines, contributing to a more nuanced understanding of how older adults continue to engage with texts. This underscores the importance of examining these dynamics in the context of very late life, where the evolution of cognitive processes and their influence on reading comprehension remain underexplored.

### 1.3. The Present Study

It is known that cognitively healthy older adults, particularly those in the third-age group, maintain reading comprehension skills and exhibit similar performance in several aspects of reading comprehension processing to those found in young adults. However, studies on the fourth-age group are almost non-existent. As a result, it remains unclear whether changes in reading comprehension skills occur after the age of 80. Nonetheless, evidence in other linguistics tasks, such as sentences comprehension (Stewart and Wingfield 2009) and lexical production (Rojas et al. 2023a, 2023b), suggests that a noticeable decline is observed after this age. In this context, the aim of the present study is to determine whether there are differences in reading comprehension performance between the third-age group (60–79) and the fourth-age group (80 and over), and how cognitive functioning, sex, reading habits, and formal education level influence their performance.

## 2. Methods

### 2.1. Participants

A sample of 150 monolingual older adults voluntarily participated in this study. Recruiting participants aged 60 and above, particularly those over 80, presents significant challenges due to health, mobility, and availability constraints. Despite these obstacles, we successfully enrolled 150 participants meeting strict inclusion and exclusion criteria, comprising 86 individuals in the third-age group (60–79 years) and 64 in the fourth-age group (80+ years). While larger sample sizes are generally preferred for enhancing statistical power, studies focusing on these age groups often work with smaller samples due to similar recruitment challenges (e.g., Martín-Aragoneses et al. 2023; Artuso and Belacchi 2021; Rojas et al. 2023a).

The sample was divided into two groups according to the age of the participants: third-age group: 60–79 years; and fourth-age group: 80–90 years. All older adults were recruited through the “Más Adultos Mayores Autovalentes”, sponsored by the Chilean government. Inclusion criteria were as follows: 60 years of age or older, at least 6 years of schooling, normal (or corrected) hearing and vision, urban residence, and completion of the reading comprehension task within a maximum of two weeks. Table 1 presents descriptive demographic statistics of our sample.

In addition, participants’ medical records had to be kept up to date to ensure that they were in good health. We also set the following exclusion criteria: a history of cerebrovascular disease, a diagnosis of neurodegenerative disease, depression or psychiatric illness, and finally, a risk score on any of the following psychometric tests: Mini-Mental State Evaluation (MMSE score < 23 points; Folstein et al. 1975), Montreal Cognitive Assessment (MoCA score < 21 points; Delgado et al. 2019), or Geriatric Depression Scale-15 (score > 11 points; Martínez de la Iglesia et al. 2002; Ortega et al. 2007). Approximately 210 older people were invited to participate. We set a sample size that could provide at least 3000 data points. Of those interested in participating, older adults who did not meet the inclusion and/or exclusion criteria were excluded.

To participate in this study, all the older individuals read and signed an informed consent, approved by the Ethics Committee of the sponsoring University. The objectives and details of the study were presented to the authorities of each club (university-associated senior clubs). Then, older adults interested in participating were assessed for cognitive (MMSE and MoCA) and emotional performance (Geriatric Depression Scale-15). Finally, the selected individuals were invited to the University’s Specialty Laboratory (or the office of attention’s more self-supporting older person program) to perform a reading comprehension test, and a reading habit and self-perceived reading difficulties questionnaire for older persons.

### 2.2. Materials and Design

#### 2.2.1. Reading Comprehension for Older Adults’ Task

The reading comprehension task for older adults (see Supplementary Material S1) consisted of 4 short texts and a total of 26 multiple-choice questions (Text 1 = 6 questions; Text 2 = 7 questions; Text 3 = 7 questions; and Text 4 = 6 questions). The task had 4 different forms, in which only the order of the texts was reversed. The texts and their corresponding questions were based on Kintsch’s psycholinguistic model (Kintsch 1998; Kintsch and Rawson 2005). The 26 items asked about different aspects of text content at local and global levels, as well as implicit and explicit information. The texts were 607, 479, 557, and 610 words long. Two texts were descriptive, one was a media text, and one was a short story. Each multiple-choice question presented four alternatives, of which only one was correct (one point per correct alternative). To confirm the complexity and length of the task in the population of interest, a pilot study was conducted with 10 older adults (different from those selected for the final sample). The reliability analysis of the reading comprehension questionnaire produced a Cronbach’s alpha of 0.9, indicating good internal consistency. The 95% confidence interval for the alpha ranged from 0.84 to 0.95. These findings support the reliability of the scale for effective measurement of reading comprehension in older adults.

#### 2.2.2. Reading Habits and Reading Difficulties Self-Image Questionnaire for Older Adults

To develop this questionnaire (see Supplementary Material S2), we used the Chilean government’s study on reading behavior at the national level (https://www.cultura.gob.cl/estudios/observatorio-cultural; accessed on 4 March 2021). We selected and adapted only those questions that were aimed at assessing the reading habits and self-image with respect to reading difficulties of older people. Our questionnaire contained 17 multiple-choice questions. There were 10 questions on reading habits and 7 questions on self-image with respect to reading difficulties. The questions on reading habits presented four response alternatives (never, 0 points; a few times, 1 point; often, 2 points; always, 3 points), and the participants had to choose only one. The questions on the self-perception of reading difficulties presented 5 alternatives (never, 0 points; rarely, 1 point; sometimes, 2 points; often, 3 points; always, 4 points). To corroborate the length and clarity of the questions in the population of interest, a pilot study was conducted with 10 older adults (different from those selected for the final sample). Only the wording of two questions on self-perceived reading difficulties was adjusted. The reliability global analysis (all 17 items) of the questionnaire yielded a Cronbach’s alpha of 0.98 with CI95% equal to 0.96 and 0.99, indicating excellent internal consistency. The reliability analysis by component—that is, habits and self-image—yielded the same results (Cronbach’s alpha = 0.99 and 0.98, respectively).

### 2.3. Procedure

Cognitive (MMSE and MoCA) and emotional (Geriatric Depression Scale-15) assessments were administered in an initial 45-min session. Participants were summoned to the Language and Cognition Laboratory of the sponsoring university. A specialist interviewed the participants and administered the tests in a specially adapted procedure room (acoustically isolated, lit, and heated). Participants who scored above the cut-off criteria of the cognitive assessment and below the emotional assessment on each of the tests were invited to a second session to assess reading comprehension and reading skills. Participants who did not meet the cut-off scores were invited to a more detailed cognitive assessment and to cognitive stimulation workshops at the sponsoring university. The reading comprehension task and the questionnaire on reading habits and self-image with respect to reading difficulties were administered in the second session in the same room of the Language and Cognition Laboratory of the sponsoring university. Both tasks were administered during the same session, although in some cases, they were completed within a week of each other.

The reading comprehension task was presented in digital format on a 15-inch high-resolution laptop. The four texts were displayed randomly, one at a time, each followed by its respective multiple-choice questions. All participants were instructed to read each text at their own pace and then select the alternative they considered correct. They could also refer back to the text if necessary. Participants were free to scroll through the digital text and select the chosen alternative using the mouse. Additionally, the evaluator helped in case of difficulties in using the mouse or other technical obstacles. Each participant’s responses were automatically recorded in an electronic memory. The test took about 30 to 40 min.

The questionnaire on reading habits and self-image with respect to reading difficulties was administered in printed form. The 10 questions on reading habits were presented first, followed by the 7 questions on self-perceived reading difficulties. The evaluator read each question and its alternative out loud. They were instructed to choose the alternative that best represented them, and it was made clear that there were no right or wrong answers. In addition, the evaluator could clarify doubts and questions or repeat the questions and alternatives if necessary. Each participant’s answers were recorded by the evaluator. The questionnaire took about 15 min to complete.

### 2.4. Data Analysis

**Figure 1:**
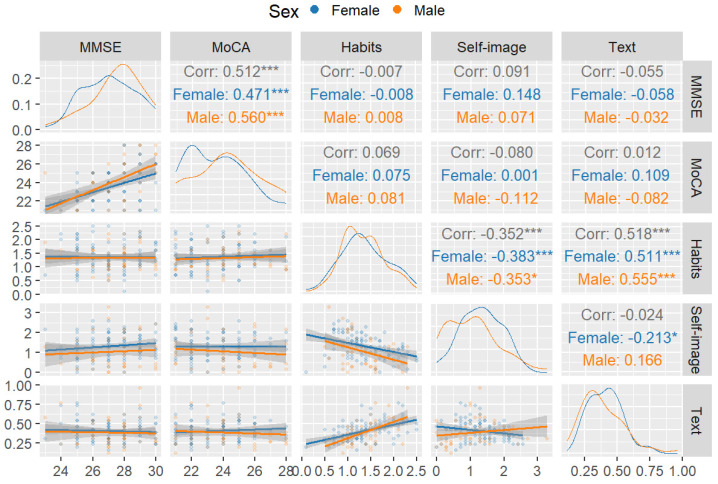
Correlogram for the MMSE, MoCA, habits, self-image, and text comprehension divided by sex (Note: * = *p* < .05, *** = *p* < .001).

**Figure 2:**
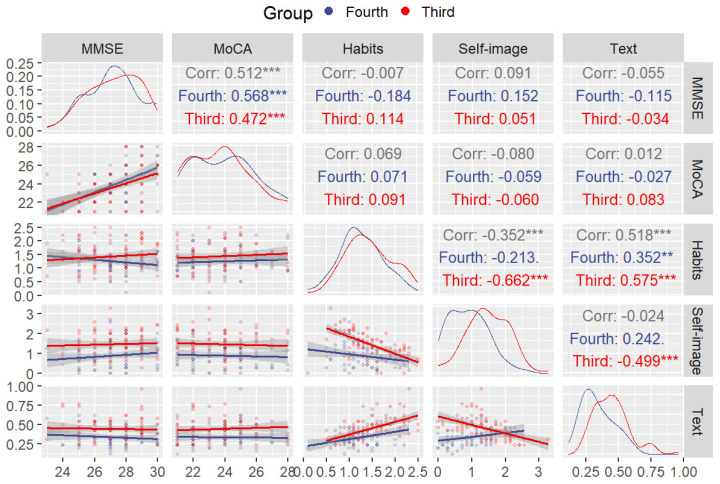
Correlogram for the MMSE, MoCA, habits, self-image, and text comprehension divided by age group (third and fourth age). (Note: ** = *p* < .01, *** = *p* < .001).

To report the results of our study, we use a combination of descriptive (see Table 2) and inferential statistics (see Table 3). At the descriptive level, we explored the relationship between all the relevant variables. For this purpose, we used correlograms as they are a powerful graphical tool for visualizing and describing the relationships between multiple variables. In our study, the correlogram included MMSE, MoCA, habits, self-image, and text comprehension and was divided by sex (Figure 1) and by age group (Figure 2). This allowed us to observe how relationships between variables might differ between sex and/or age groups. We also included linear model fits to allow us to visually assess the linear relationships between pairs of variables, providing a clear representation of trends and potential correlations. Finally, the top triangle shows the correlation coefficients. These coefficients are crucial in quantifying the strength and direction of the linear relationships between variables.

Finally, to assess the effect of these variables on adults’ text comprehension, we used a generalised mixed-effects binomial regression (Baayen et al. 2008). These models are a combination of fixed and random effects. The fixed effects in our regression include sex, group, MMSE, MoCA, education, habits, and self-image scores, all of which were centred and scaled to avoid multicollinearity. We also included random intercepts for participants and items in the model. Random effects account for variation between participants and items, recognizing that there may be variability in the data that are not captured by the fixed effects alone. This approach is particularly relevant to psycholinguistic datasets where the assumption of independence between observations is untenable (see Clark 1973).

To evaluate the statistical power, we followed a simulation-based power analysis approach (Brysbaert and Stevens 2018; Kumle et al. 2021). Specifically, we generated 1000 simulated datasets reflecting the observed probabilities of correct responses in our reading comprehension task. After that, we fitted the same generalized mixed-effects binomial regression used for the analysis of our actual data for each simulated dataset. These models included fixed effects for sex, age group, MMSE, MoCA, schooling, reading habits, and self-image scores, along with random intercepts for participants and items.

## 3. Results

Table 2 shows descriptive statistics for all measures. Figure 1 shows the first correlogram presenting the correlations between the MMSE, MoCA, habits, self-image, and text comprehension, divided by sex. The histogram on the diagonal illustrates the distribution of each variable, while the lower triangle presents scatterplots with fitted regression lines, indicating the direction and strength of linear relationships. The upper triangle quantifies these relationships with correlation coefficients, where the significance of correlations is marked with asterisks.

The cognitive assessments, MMSE and MoCA, yielded a significant positive correlation, with r = 0.560 (*p* < .001; d = 1.35) in men and r = 0.471 (*p* < .001; d = 1.06) in women, suggesting a strong link between these cognitive measures across sex. Habits were significantly inversely correlated with self-image scores, both in men, r = −0.353 (*p* < .05; d = .75) and women, r = −0.383 (*p* < .001; d = .82). Instead, habits displayed a positive correlation with text comprehension in men (r = 0.555; *p* < .001; d = 1.33) and women (r = 0.511; *p* < .001; d = 1.18). Finally, self-image about reading difficulties was negatively correlated with text comprehension skills, but only in women (r = −0.213; *p* < .05; d = .43).

Figure 2 shows the second correlogram presenting the same variables but divided by age group (third and fourth age) rather than sex. As in the previous correlogram, MMSE and MoCA correlate in both age groups (third-age: r = 0.472; *p* < .001; d = 1.07, and fourth-age: r = 0.568; *p* < .001; d = 1.38). Moreover, we observed a negative correlation between habits and self-image, which reached significance only for the third-age group (r = −0.662; *p* < .001; d = 1.76). Similarly, text comprehension skills were significantly negatively correlated with self-image, but again, only for the third-age group (r = −0.499; *p* < .001; d = 1.15). Finally, text comprehension skills were positively with reading habits for both the third- (r = −0.575; *p* < .001; d = 1.40) and the fourth-age group (r = −0.352; *p* < .01; d = 0.75).

The results from the generalized linear mixed model (see Table 3) revealed that reading habits had a statistically significant positive influence on the text comprehension skills (β = 0.281; se = 0.048; z-value = 5.80; *p* < 0.001). Moreover, participants in the third-age group had significantly higher scores in the text comprehension test (β = −0.145; se = 0.053; z-value = −2.72; *p* < 0.01). Other variables, including MoCA, MMSE, schooling, sex, and self-image, did not appear to significantly modulate text comprehension skills.

Finally, the simulations evidenced that the effect of reading habits exhibited full power (100%), indicating that our dataset was robust enough to detect differences associated with this variable. In contrast, the effect of age group reached a power of 60%, suggesting a relatively low yet directionally valid capacity to detect differences between younger and older participants. The effects of the remaining predictors showed substantially lower power: self-image (2.8%), MMSE (2%), MoCA (7.4%), age of schooling (4%), and sex group (<1%). Although the inclusion of these variables contributes to the explanatory comprehensiveness of the model, their extremely low power indicates that these effects are so small that they will require an unrealistic sample size for clearer detection; in other words, these effects are neglectable. Overall, these simulations confirm that the study design effectively identifies the influence of reading habits on text comprehension and provides preliminary support for the role of age group, albeit with moderate statistical power.

## 4. Discussion and Conclusions

The aim of this study was to explore potential differences in reading comprehension performance between individuals in the third age (60–79 years) and the fourth age (80 years and older), as well as to examine how cognitive functioning, sex, reading habits, and level of formal education influence their performance. To achieve this, we examined the effects of age, sex, cognitive functioning, educational level, and reading habits on reading comprehension among older adults, distinguishing between those in the third age and the fourth age. Our results reveal a notable decline in reading comprehension abilities as individuals transition from the third to the fourth age, underscoring the influence of advanced aging on written language understanding. Furthermore, this study highlights the significant role of reading habits in sustaining reading comprehension. Specifically, strong reading habits were associated with better reading comprehension performance. This was true for both sex and age groups, as can be seen in the correlograms, suggesting that engagement in reading across the lifespan, with its contribution to improve knowledge reserves as crystallized intelligence, may be associated with a reduction in the cognitive decline typically observed in later life stages.

Our results indicate that older adults with consistent reading habits demonstrate better reading comprehension skills in both age groups. This supports the idea that regular reading equips older adults to handle complex texts more effectively. Moreover, our findings underscore the positive effects of reading habits on reading comprehension, even in advanced age, aligning with the cognitive reserve theory (Cooley 2020). This theory posits that engaging in intellectually stimulating activities, such as regular reading, builds a reserve of cognitive skills that helps to mitigate the influence of age-related cognitive decline. Thus, sustained reading habits may enhance cognitive resilience, enabling older individuals to maintain higher levels of language processing and comprehension despite aging-related cognitive challenges (Bavishi et al. 2016; Rodrigues et al. 2021; Malcorra et al. 2022b).

Our findings align with those of Freed et al. (2017), who identified language experience as one of the primary predictors of reading comprehension in adults. The significant role of reading habits observed in our study suggests that accumulated linguistic experience continues to support reading comprehension in older adults, potentially compensating for declines in cognitive functions such as working memory. Furthermore, strong reading habits may also mitigate declines in other cognitive areas, which could be explained by the complexity and interconnectedness of cognitive constructs in reading comprehension, as proposed by Goring et al. (2021). This emphasizes the need for holistic approaches when studying reading comprehension among older adults, acknowledging the complex relationship between cognitive abilities and linguistic experience.

Interestingly, cognitive skills, within a non-impaired range, indicative of higher fluid intelligence, did not significantly influence reading comprehension in any group. This finding, along with the effect of reading habits, which reflects accumulated experience in reading, suggest that knowledge and skills gained over time—i.e., crystallized intelligence—remain relatively stable in both the third and the fourth age. Thus, beyond the cognitive reserve fostered by reading habits, frequent reading activity may provide a compensatory mechanism for potential declines in fluid intelligence, such as processing speed and working memory, which are critical for language processing tasks. Therefore, even as fluid intelligence diminishes, the retention and application of crystallized intelligence likely allowed older adults to maintain functional reading skills (Riffo et al. 2020; Stine-Morrow et al. 2016; Ng et al. 2019; Malcorra et al. 2022a). This highlights a crucial interplay between different types of cognitive abilities in mitigating the influence of aging on reading comprehension.

Surprisingly, educational level did not show an effect on reading comprehension. This finding does not align with existing literature on the role of formal education in enhancing text comprehension (see, e.g., Kavé et al. 2012). This divergence may stem from the educational composition of our participant pool, which predominantly included individuals with lower educational levels. This characteristic reflects broader demographic trends in Chile, as noted in the methods section of our article. Specifically, over 70% of individuals aged 60 and older have not completed primary school education, a consequence of the limited coverage provided by the Chilean educational system prior to the 1960s—the era when many of our study participants were born. This context may present a limitation in our study as it affects the generalizability of our findings across populations with varying educational backgrounds. Nevertheless, the results suggest that in populations with lower educational levels, reading habits may help compensate for educational deficits, playing a key role in supporting reading skills (Malcorra et al. 2022a).

An intriguing aspect of the results of the present study is the negative correlation between self-perceived difficulties and reading comprehension. That is, the lower self-perceived the difficulty, the greater the performance in reading comprehension. However, the correlation was only significant in the third age and in the female groups. Self-image with respect to difficulties might reflect a metacognitive skill. Therefore, this result might reflect diminished metacognitive skills in men of the fourth-age group. Considering that no sex differences were found in all the other analyses, this finding might also reflect a greater heterogeneity composition of the older age group, as reported by Margrett et al. (2016). It is unclear whether the observed behavior in older men is more influenced by aging itself or by specific cultural and generational factors. Further research is required to pinpoint specific factors that may explain the differences observed in this study.

Our findings provide valuable insights for designing educational programs tailored to the cognitive and linguistic needs of older adults. To enhance and maintain reading comprehension, educational interventions should emphasize the development of consistent reading habits (Sörman et al. 2018; Ng et al. 2019; Chang et al. 2021). Furthermore, the observed role of reading habits in text comprehension among older adults carries important implications for the general population. Our results suggest that establishing strong reading habits from a young age can lay a foundation for cognitive benefits that extend throughout the lifespan. In schools, promoting a culture of regular reading could serve as a preventive strategy against cognitive decline or as a way to accumulate knowledge and experience to compensate for it. In this sense, our research highlights the broader implications of reading habits as an activity that supports sustained comprehension skills across the lifespan.

To build upon the findings presented, future research should consider conducting longitudinal studies that trace the evolution of reading habits and comprehension across different stages of aging, particularly during the transition between the third and the fourth age. Such studies could provide valuable insights into the progressive changes in comprehension capabilities. Additionally, exploring the interaction between reading habits and other cognitive abilities could help delineate the specific contributions of other intellectual skills to cognitive resilience. Gaining a deeper understanding of these dynamics can inform targeted interventions that promote cognitive health and literacy skills, ultimately enhancing quality of life among the elderly. Finally, for a more in-depth study of the influence of the educational level on the reading comprehension of older people, it would be necessary to conduct a study that includes a population with higher levels of education than those considered in this article.

## Figures and Tables

**Table 1:** Demographics by age group and sex.

Group	Sex	n	Mean	Age	Min|Max	Mean	Schooling^1^	Min|Max
				sd			sd	
3rd	Male	63	69.17	4.99	62|78	11.48	1.34	6|12
3rd	Female	23	68.44	4.94	60|79	10.97	1.77	6|12
4th	Male	35	83.62	2.18	80|88	9.07	2.66	6|12
4th	Female	29	84.17	3.01	80|90	8.74	2.37	6|12

**Table 2:** Descriptive statistics for the MMSE, MoCA, habits, self-image, and text comprehension variables.

	Min	Median	Mean	Sd	Max
MMSE	23,000	27,000	27,210	1.672	30,000
MoCA	21,000	24,000	23,750	1.925	28,000
Habits	0.100	1.300	1.359	0.479	2.500
Self-image	0.000	1.286	1.230	0.672	3.286
Text Comprehension	0.115	0.385	0.399	0.142	0.962

**Table 3:** Generalized mixed-effect regression results.

	Estimate	SE	z-Value	*p*-Value
(Intercept)	−0.496	0.116	−4.28	0.000 ***
MMSE	−0.087	0.047	−1.85	0.064
MoCA	0.053	0.048	1.12	0.264
Schooling	0.051	0.047	1.09	0.277
Sex	0.009	0.044	0.21	0.831
Age	−0.145	0.053	−2.72	0.007 **
Habits	0.281	0.048	5.80	0.000 ***
Self-image	0031	0.052	0.58	0.559

*** = *p* < .001; ** = *p* < .01.

## Data Availability

All data and scripts for the analysis can be found at https://osf.io/48hkc/?view_only=a7069b583f6947a9b5030aa366c5dc89.

## Note

1 According to the Chilean National Institute of Statistics (2017), 70.3% of men and 76% of women older than 60 have an incomplete primary school education.

## Supplementary Materials

The following supporting information can be downloaded at https://www.mdpi.com/article/10.3390/jintelligence13010004/s1: Supplementary S1: Reading comprehension for older adults’ task; Supplementary S2. Reading habits and reading difficulties self-image questionnaire for older adults.
