# Supplementary material for: Reading Comprehension in Older Adults—Effects of Age, Educational Level, and Reading Habits
Source: J Intell. 2024 Dec 31;13(1):4. doi: 10.3390/jintelligence13010004 (PMC11765946; doi:10.3390/jintelligence13010004)
Supplement: Supplementary file 1 [file jintelligence-13-00004-s001.zip › SM1. Reading comprehension for older adultsí» task (Form 2).pdf]

PRUEBA DE COMPRENSIÓN LECTORA PARA PERSONAS MAYORES

* Indica que la pregunta es obligatoria

1. Nombres y Apellidos *

2. Rut *

Texto 4

Científicos japoneses curan Parkinson en monos con células madre humanas

- Equipo de la Universidad de Kyoto logró eliminar temblores. Al cabo de un año, continúa la mejoría en los animales.

- Técnica usa células madre embrionarias que, trasplantadas en el cerebro, se convierten en neuronas productoras de dopamina.

- La enfermedad de Parkinson se produce por la destrucción de las neuronas de la sustancia nigra. Ellas son las encargadas de producir dopamina, un neurotransmisor que controla los movimientos del cuerpo. Cuando faltan, aparecen temblores y rigidez.

Un importante paso en conseguir la cura del Parkinson fue anunciado ayer por un equipo de científicos japoneses. En experimentos con primates, los investigadores lograron borrar los síntomas de la patología, que hoy afecta a unos cuatro millones de personas en el mundo, usando células madre embrionarias de humanos. Ya en 2005, el científico Jun Takahashi, de la Universidad de Kyoto, había conseguido algo parecido: frenar la enfermedad en animales, pero en esa oportunidad había utilizado células madre embrionarias de monos.

Como se sabe, estas células tienen la capacidad de convertirse en tejido especializado de cualquier parte del cuerpo. Y la idea es utilizar esta habilidad para que se transformen en neuronas productoras de dopamina. Este neurotransmisor, encargado de controlar el movimiento muscular, ha sido sindicado como una de las principales causas del mal de Parkinson: en los pacientes con esta patología disminuye la presencia de dopamina en el cerebro y este no es capaz de enviar las señales en forma adecuada. Es cuando surgen los temblores involuntarios, la pérdida de equilibrio y la rigidez muscular, un daño que se vuelve crónico en el tiempo.

En noviembre del año pasado, un equipo estadounidense había usado una técnica similar, con resultados muy parecidos. Pero, en esa oportunidad, las células madre habían sido inducidas en laboratorio a convertirse en neuronas, antes de ser inyectadas. Ahora, lo que Takahashi logró fue que las células madre embrionarias

se transformaran en neuronas productoras de dopamina dentro del cerebro de los primates. De hecho, el 65% de las células inyectadas eran madre embrionarias y sólo el 35% eran inducidas en laboratorio. El éxito fue completo: las nuevas neuronas generaron el químico y crearon suficientes conexiones.

Al cabo de un año de seguimiento, el 10% de las nuevas neuronas se mantenían y eso era suficiente para que los animales siguieran sin presentar síntomas. “La mejoría fue evidente en sus movimientos”, dijo Takahashi a la agencia AFP.

Humanos

Ahora bien. El uso de células madre embrionarias de humanos, aunque sea en monos, es un tema crucial en esta investigación. Y no solo porque es una forma de probar su capacidad de convertirse en el tipo de neurona que se necesita. También para evitar la formación de tumores. Uno de los riesgos que existen en este tipo de terapias es que las células que se trabajan en laboratorio y luego se inyectan presenten un crecimiento descontrolado, fenómeno que origina los tumores. Pero en el experimento de Takahashi no hubo señales de este proceso. Al cabo de un año, los primates seguían sanos.

El objetivo de los investigadores japoneses ahora es lograr que el 70% de las nuevas neuronas sobreviva y, luego, probar esta técnica en un estudio clínico en humanos. Para Marcelo Miranda, neurólogo experto en Parkinson de la Clínica Las Condes, estas investigaciones son promisorias para la búsqueda de una cura. Sin embargo, prefiere ser cauteloso: “Aún falta trabajo por desarrollar”, dice. El equipo japonés también se encuentra trabajando con células madre pluripotenciales (que se obtienen de tejidos adultos de piel) y esperan obtener resultados similares. Una forma de evadir el cuestionamiento ético que en muchos países surge con la utilización de células embrionarias.

Fuente: Diario La Tercera.

3. 21. La sustancia nigra es: *

Marca solo un óvalo.

- a) la estructura cerebral encargada de la producción de dopamina.
- b) la zona cerebral caracterizada por su coloración oscura.
- c) el conjunto de células madre embrionarias que regulan la producción de dopamina.
- d) la estructura cerebral encargada del control de los movimientos musculares.

4. 22. ¿Qué es una célula madre embrionaria? *

Marca solo un óvalo.

- a) Célula que puede dar origen a un organismo completo.
- b) Célula especializada en cumplir una determinada función.
- c) Célula neuronal capaz de producir dopamina.
- d) Célula que da origen a los distintos tejidos de un organismo.

5. 23. De acuerdo con lo informado en el texto, la probabilidad de que la nueva terapia fracase podría deberse a que *

Marca solo un óvalo.

- a) la manipulación de las células madre en el laboratorio sea inadecuada.
- b) la producción de dopamina sea insuficiente para lograr el efecto deseado.
- c) se produzca un rechazo de las células trasplantadas en el cuerpo del paciente.
- d) se produzca un crecimiento descontrolado de las células trasplantadas.

6. 24. Según el texto, Takahashi utilizó en una de sus investigaciones células madre embrionarias de humanos, porque *

Marca solo un óvalo.

- a) se regeneran y multiplican rápidamente.
- b) pueden transformarse en el tipo de tejido que se requiere.
- c) reducen el riesgo de formación de tumores.
- c) resisten mejor ciertas condiciones adversas.

7. 25. Según lo que se informa en el texto, ¿cuál es el orden cronológico de los avances vinculados a la investigación del Parkinson? *

I. Takahashi y su equipo logran curar el Parkinson en monos implantando en su cerebro células madre embrionarias de seres humanos.
II. Takahashi y su equipo logran detener la enfermedad en animales usando células madre embrionarias de monos.
III. Investigadores japoneses trabajan para conseguir una mayor sobrevivencia de las células generadas artificialmente.
IV. Investigadores norteamericanos intervienen células madre en el laboratorio para convertirlas en neuronas productoras de dopamina.

Marca solo un óvalo.

- a) III-IV-I-II.
- b) II-IV-I-III.
- c) IV-III-II-I.
- d) I-IV-II-III.

8. 26. ¿Cuál de las siguientes afirmaciones es más pertinente como valoración ética de la investigación realizada por el equipo japonés? *

Marca solo un óvalo.

- a) El alto costo de la investigación en biotecnología limita enormemente la posibilidad de que los paises pobres se beneficien de sus resultados.
- b) Para obtener resultados definitivos, el equipo tendrá necesariamente que probar sus técnicas en humanos, lo que parece muy peligroso.
- c) Si bien los resultados obtenidos por el equipo japonés son auspiciosos para la cura del mal de Parkinson, es objetable en ella el uso de embriones humanos.
- d) La investigación médica orientada a buscar la cura a enfermedades catastróficas debería poder realizarse sin cuestionamientos éticos que limiten su desarrollo.

Texto 3

Sobreabundancia y hambre

En los países subdesarrollados más de 850 millones de personas sufren de desnutrición, es decir, no reciben un aporte energético suficiente para mantener un peso constante y efectuar una actividad física. Sobre todo por falta de carbohidratos, el consumo diario de calorías de los habitantes de estas regiones es inferior al mínimo indispensable, estimado en alrededor de 2.500 calorías por persona.

Otros 2.000 millones de personas están mal alimentadas: su dieta carece de proteínas, vitaminas y minerales. Por el contrario, en los países ricos, la alimentación es muy variada y se consume un exceso de calorías, alrededor de 3.000 diarias.

Las causas del hambre

La alimentación insuficiente de la población de los países subdesarrollados no deriva de limitantes naturales o de su gran crecimiento demográfico. De hecho, en las últimas décadas el crecimiento natural de la población ha sido inferior al aumento de la producción agrícola. Se ha visto que, a nivel mundial, la producción de alimentos, sobre todo la de cereales, es suficiente para alimentar a toda la población de la Tierra.

La causa principal del hambre y de la mala nutrición masificada en los países subdesarrollados se encuentra en el bajo poder adquisitivo de las personas, es decir, en la escasa cantidad de bienes que puede comprar cada habitante. Los que viven en áreas rurales o en las poblaciones marginales de las grandes ciudades no tienen el dinero suficiente para comprar alimentos o no cuentan con los medios para cultivarlos. Una segunda razón reside en el grado de abandono en que se encuentra la agricultura de subsistencia en los países subdesarrollados. Sin los adecuados programas de incentivo, los cultivos de subsistencia –por ejemplo, los cereales– terminan por ser abandonados, dejando lugar a las plantaciones de las multinacionales occidentales. Por otro lado, los países pobres entran en un círculo vicioso: venden los productos de sus plantaciones a los países ricos y se ven obligados a adquirir los alimentos de primera necesidad a precios más altos.

La sobreabundancia

En los países desarrollados el principal problema, en cambio, lo constituye la producción excesiva de alimentos con respecto a las necesidades de los consumidores. Estados Unidos y la Unión Europea se ven obligados a financiar, con subsidios, a los agricultores que no alcanzan a vender todos sus productos. Para no vender sus excedentes -de cereales en Estados Unidos o de mantequilla en la Unión Europea- a precios muy bajos, los países desarrollados prefieren acumularlos, a pesar de los elevados gastos de conservación.

La "revolución verde"

A partir de la década de 1960, algunas organizaciones internacionales como la FAO han financiado en muchos países del tercer mundo programas de riego y de innovaciones tecnológicas, promoviendo el cultivo de cereales de alto rendimiento. Aplicando los conocimientos de ingeniería genética -las técnicas que modifican las características de los organismos vivos-, se han introducido nuevas variedades de maíz en México, de arroz en la India, y de trigo en África septentrional. El objetivo es aumentar la producción para permitir que estos países alcancen la autosuficiencia alimentaria.

De hecho, en esas áreas se ha logrado un crecimiento en las cosechas agrícolas. Sin embargo, el conjunto de técnicas y de innovaciones, que reciben el nombre de "revolución verde", no ha resuelto el problema del hambre en el mundo. Pocos agricultores las han introducido, ya que requieren de modernas tecnologías e importantes capitales.

FUENTE: *Enciclopedia Escolar Larouse*.

9. 14. La "revolución verde" se define en el texto como *

Marca solo un óvalo.

- a) el crecimiento que ha experimentado la agricultura desde la década del ´60.
- b) la producción a gran escala de cereales en países con agricultura subdesarrollada.
- c) un movimiento que promueve la producción agrícola libre de contaminación.
- d) un conjunto de técnicas que permiten fomentar y mejorar la producción agrícola.

10. 15. De acuerdo con lo informado en el texto, la FAO es una organización que se ha preocupado de *

Marca solo un óvalo.

- a) resguardar la salud de los habitantes de los países subdesarrollados.
- b) promover el uso de tecnologías en la producción de alimentos.
- c) mejorar el poder adquisitivo de los habitantes del tercer mundo.
- d) criticar las políticas de los países desarrollados en materia de alimentación.

11. 16. ¿Cómo se han producido en México e India nuevas variedades de maíz, arroz y trigo? *

Marca solo un óvalo.

- a) Realizando un proceso de manipulación genética de las semillas de cada especie.
- b) Aplicando programas especiales para optimizar el riego y la fertilización.
- c) Desarrollando modernos sistemas de ingeniería para combatir las plagas.
- d) Creando métodos que favorecen su cultivo en condiciones ambientales adversas.

12. 17. La información del texto se organiza en torno a dos sub-temas: *

Marca solo un óvalo.

- a) las causas del problema que experimentan los países subdesarrollados y la innovación de la ingeniería genética.
- b) definiciones de los principales conceptos involucrados en el tema y el aporte de la revolución verde.
- c) la comparación entre países desarrollados y subdesarrollados, y una posible solución al problema.
- d) cifras que describen las precarias condiciones alimentarias del tercer mundo y algunas conclusiones sobre el tema.

13. 18. En relación con la sobreabundancia y el hambre, el autor del texto se propone principalmente *

Marca solo un óvalo.

- a) presentar un punto de vista ecológico sobre el tema.
- b) exponer el problema explicando sus causas.
- c) discutir sus aspectos humanitarios y éticos.
- d) formular una crítica considerando aspectos sociales y políticos.

14. 19. El término "subsidio" que aparece en el texto hace referencia a *

Marca solo un óvalo.

- a) asistencia técnica.
- b) préstamo bancario.
- c) impuesto fiscal.
- d) apoyo económico.

15. 20. En los países subdesarrollados, el aporte energético de la alimentación diaria por habitante *

Marca solo un óvalo.

- a) está por debajo del mínimo esencial.
- b) se encuentra al mismo nivel que en los países ricos.
- c) se ubica en el nivel adecuado.
- d) alcanza aproximadamente las 2.500 calorías.

Texto 2

La máquina voladora

En la mañana del segundo mes del año 400 de nuestra era, el emperador Yuan bebía un poco de té y se abanicaba protegiéndose del calor cuando un sirviente cruzó el jardín gritando:

- Emperador, emperador ¡un milagro!
- ¿Qué dices? – preguntó el emperador.
- Lo vi en el aire, con alas. Oí una voz que venía del cielo. Era un dragón con un hombre en la boca, un dragón de papel y bambú.
- Qué extraño me parece -dijo el emperador- . Vamos y muéstrame lo que has visto.

Se internaron ambos en el jardín y subieron luego a una colina. El emperador miró el cielo y vio muy arriba a un hombre riéndose. Estaba vestido con papeles brillantes, cañas como alas y una hermosa cola amarilla. Volaba de un lado a otro como un pájaro, como un dragón en una región de dragones.

- ¡Vuelo! ¡Vuelo! –gritaba el hombre.

El sirviente lo saludó, pero el emperador Yuan no se movió. Miró la gran muralla sobre las verdes colinas, la maravillosa muralla que los protegía desde tiempos inmemoriales de las hordas enemigas y había preservado la paz durante tantos años.

El emperador miró el cielo otro minuto y dijo:

- Dile que baje.
- ¡Eh, baja, baja! ¡El emperador quiere verte!

El hombre volador bajó deslizándose con un susurro de papeles y bambúes. Se acercó orgulloso y se inclinó ante el emperador.

- ¿Qué has hecho? –preguntó el emperador.
- He volado por el cielo, excelencia.

- ¿Qué has hecho? –dijo otra vez el emperador.
- Acabo de decirlo, señor.
- No me has dicho nada.

El sol estaba alto en el cielo ahora. El emperador, el sirviente y el hombre se encaminaron al palacio. Al llegar, el emperador llamó a los guardias, los que vinieron corriendo.

- Apresad a este hombre y llamad al verdugo –ordenó el emperador.

Los guardias cumplieron la orden.

- ¿Qué he hecho? –gritó el hombre alado y se puso a llorar.
- Este hombre ha inventado una máquina –dijo el emperador- y nos pregunta qué ha hecho. El mismo no lo sabe. Ha inventado sin saber para qué servirá su invento.
- He descubierto belleza –dijo el hombre. Me he deslizado por el aire como un pájaro. Me he sentido libre.
- Sí –dijo con tristeza el emperador. Así es. Sentía que mi corazón palpitaba contigo en el aire.
- Entonces, perdóname la vida.
- A veces, -dijo más tristemente el emperador- debemos renunciar a pequeñas bellezas para conservar las que tenemos. No te temo a ti. Temo a otro hombre que hará una máquina como la tuya. Este hombre tendrá un corazón malvado y la belleza habrá desaparecido. No volará ya en un aparato de papel y cañas y arrojará enormes piedras sobre la gran muralla.

Nadie se movió ni habló.

- Cortadle la cabeza –dijo el emperador.

Ray Bradbury (adaptación), 1953.

16. 7. El emperador miró la gran muralla, porque *

Marca solo un óvalo.

- a) ella los había protegido desde siempre de las hordas enemigas.
- b) relacionó el invento con la pérdida de seguridad para la nación.
- c) deseaba contemplar la belleza y majestuosidad de la gran muralla.
- d) pensó que con la máquina inventada se podría destruir la gran muralla.

17. 8. El "otro hombre" al que se refiere el emperador podría ser *

Marca solo un óvalo.

- a) un aviador.
- b) un espía.
- c) un adversario.
- d) un inventor.

18. 9. ¿De qué forma contribuía la gran muralla a mantener la paz? *

Marca solo un óvalo.

- a) Detenía los ataques de fuerzas enemigas.
- b) Daba protección al palacio del emperador.
- c) Impedía el contacto con naciones vecinas.
- d) Hacía sentirse seguros a los habitantes.

19. 10. ¿Qué mensaje quiere darnos el autor, Ray Bradbury, con este relato? *

Marca solo un óvalo.

- a) El hombre siempre tuvo el anhelo de volar.
- b) La inventiva y creatividad del ser humano no tienen límites.
- c) El progreso puede convertirse en una amenaza para el hombre.
- d) Los grandes inventos siempre han generado conflictos.

20. 11. Si nos situamos en el lugar del emperador como responsable del imperio, ¿se justifica su decisión de ordenar la muerte del inventor? *

Marca solo un óvalo.

- a) Sí, puesto que pensó primero en la paz y seguridad de la nación.
- b) No, ya que no pensó en los beneficios futuros del invento.
- c) Sí, porque el inventor actuó en forma muy irresponsable.
- d) No, pues la ejecución del inventor fue una medida precipitada.

21. 12. El invento del hombre consistía en algo muy similar a lo que hoy conocemos como *

Marca solo un óvalo.

- a) planeador.
- b) paracaídas.
- c) parapente.
- d) alas delta.

22. 13. De acuerdo con el texto, ¿qué sintió el hombre mientras volaba?

Marca solo un óvalo.

- a) Libertad.
- b) Júbilo
- c) Poder
- d) Orgullo

Texto 1

Celulares….la palabra la tienen los parlamentarios.

Columnista: Edmundo Sepúlveda Marambio.
Martes, 13 de marzo de 2012
www.elurbanorural.cl

Pongamos marcha atrás y recordemos que hace dos décadas no teníamos más remedio que usar la telefonía fija tradicional para comunicarnos a distancia. Algunos tenían el privilegio de tener el teléfono instalado en su casa, quedando el resto sujeto al llamado desde las cabinas públicas, siempre y cuando fuera poderosamente necesario.

Hoy por hoy, con la increíble propagación de la práctica telefonía móvil, los contactos con el exterior se han convertido en un vicio ineludible para satisfacción y pingües ganancias de las empresas telefónicas. Claro que ganan más. Siendo generosos, casi se podría contar con los dedos de una mano las personas que todavía no tienen un celular. Si hasta el humilde campesino y los pequeños que recién balbucean el abecedario se manejan con este aparatito. Mucho más: nuestros nietos, que ni siquiera alcanzan los 3 años, nos dan clases magistrales sobre los diferentes usos de este invento, empezando por los juegos de entretención, obviamente. Y nosotros, que con suerte sabemos llamar y recibir llamados, nos quedamos sorprendidos frente a ellos. Además, esta creación del hombre, entre otras cosas, se ha encargado de que tengamos antenas satelitales levantadas en todos los cerros y en las planicies urbanas y rurales para favorecer la señal, con el consiguiente daño para la salud y el deterioro del paisaje.

Lo peor de todo es que esta proliferación descomunal del "celu" acarrea otros bemoles como, por ejemplo, la consideración de un nuevo ítem familiar que nos gastamos en cargar el aparato o en el pago del plan. Aparte de lo anterior, la singular contaminación acústica que actualmente se produce al interior de los buses y los proletarios microbuses. Seguramente esta particularidad será una tremenda novedad para los honorables legisladores, justificadamente acostumbrados a movilizarse en

locomoción propia para llegar a tiempo a sus múltiples deberes en el parlamento, visitar su parcela electoral y sonreír para la foto en los cortes de cinta, entre otros menesteres rutinarios. Novedad, digo, porque a los representantes del pueblo que cobran dieta nunca se les verá ocupando un destartalado asiento en la locomoción colectiva.

Vayamos directo al problema: los sufridos proletarios que a diario nos trasladamos en los medios de locomoción pública para llegar al trabajo, con molestia tenemos que escuchar durante el trayecto un sinnúmero de estupideces, que obligada e inevitablemente debemos soportar de los ilustres desconocidos que nos acompañan. Ni hablar (sí hablar) cuando los diálogos se producen en forma simultánea. Peor aún, el volumen de la conversación sube en grado superlativo cuando el protagonista bordea los setenta años. En esos casos, poco menos que el vocerío se transforma en gritos, de forma tal que lo escucha hasta el pasajero que va en los últimos asientos.

En consecuencia, respetables señores parlamentarios, llegó la hora de que se trabaje en un proyecto de ley que regule el ruido molesto y ensordecedor dentro de la locomoción pública, porque es justo y necesario que las personas que van leyendo o simplemente escuchando buena música y quieren un viaje feliz y reparador no sean interrumpidos a cada segundo por expresiones como "¿dónde estái?, ¿cómo estái?", y a continuación toda la verborrea de mercado persa y ferias libres. Sí, señores legisladores. Así como antaño se eliminó fumar dentro de la locomoción colectiva y, posteriormente, se reguló el uso de la radio en la misma ("la radio del vehículo puede funcionar con volumen moderado y siempre que ningún pasajero se oponga". Decreto 212, Artículo 50), es el momento de parar el escándalo a las conversaciones inoportunas e indeseadas que se producen por celular.

23. 1. En la frase "los proletarios microbuses", la palabra destacada se aplica a *

Marca solo un óvalo.

- a) trabajadores que no poseen locomoción propia.
- b) buses que transportan a trabajadores.
- c) vehículos de la locomoción colectiva.
- d) conductores de la locomoción colectiva.

24. 2. ¿A quién se dirige el autor de la columna de opinión y con qué propósito? *

Marca solo un óvalo.

- a) A los parlamentarios para que legislen sobre el uso de celulares en la locomoción colectiva.
- b) A los dueños de celulares para que los usen de manera adecuada en los espacios públicos.
- c) A los lectores para que tomen conciencia de las molestias que puede provocar el uso del celular.
- d) A los miembros del parlamento para que regulen las ganancias de las compañías telefónicas.

25. 3. En el texto se hace alusión al Decreto 212, artículo 50, con el propósito de

Marca solo un óvalo.

- a) mostrar que ya se ha legislado para la regulación del comportamiento de las personas dentro del transporte público.
- b) reforzar la necesidad que existe hoy en día de regular el comportamiento de las personas que viajan en la locomoción colectiva.
- c) exigir a los parlamentarios que revisen la legislación vigente que regula el uso del transporte público.
- d) informar al lector acerca de dos importantes leyes relacionadas con el transporte público.

26. 4. En el texto se afirma lo siguiente:"nuestros nietos, que ni siquiera alcanzan los 3 años, nos dan clases magistrales sobre los diferentes usos de este invento".Con ello el autor quiere decir que los niños

Marca solo un óvalo.

- a) desarrollan desde pequeños la capacidad de participar en juegos virtuales.
- b) desarrollan temprano la habilidad para manipular aparatos electrónicos.
- c) pueden inventar fácilmente nuevos usos del teléfono celular.
- d) pueden explicar fácilmente los diferentes usos de un teléfono celular.

27. 5. En el texto se dice: “hace dos décadas no teníamos más remedio que…”. ¿A qué época se hace referencia?

Marca solo un óvalo.

- a) A mediados de los ‘80.
- b) A fines de los ‘80.
- c) A mediados de los ‘90.
- d) A principios de los ‘90

28. 6. ¿Con qué propósito se han instalado, según el autor del texto, antenas satelitales en distintos lugares?

Marca solo un óvalo.

- a) Mejorar el funcionamiento de los satélites para la telefonía móvil.
- b) Aumentar la cobertura y la cantidad de las comunicaciones telefónicas.
- c) Recibir y enviar señales a los satélites que circundan el planeta.
- d) Favorecer la calidad de la imagen y del audio en las comunicaciones.

Este contenido no ha sido creado ni aprobado por Google.

Google Formularios
