# Supplementary material for: Reading Comprehension in Older Adults—Effects of Age, Educational Level, and Reading Habits
Source: J Intell. 2024 Dec 31;13(1):4. doi: 10.3390/jintelligence13010004 (PMC11765946; doi:10.3390/jintelligence13010004)
Supplement: Supplementary file 1 [file jintelligence-13-00004-s001.zip › SM2. Reading habits and reading difficulties self-image questionnaire for older adults.pdf]

Cuestionario de hábitos de lectura y autopercepción de dificultades en personas mayores

PROYECTO FONDECYT 1211754

Nombre:
Edad:
RUT:

I. Hábitos de lectura

1. ¿Con qué frecuencia lee?
    - Nunca (0 punto)
    - Muy poco (1 punto)
    - Ocasionalmente 2 puntos)
    - Siempre (3 puntos)

2. Cuando usted era niño/a, ¿con qué frecuencia alguien le leía?
    - Nunca (0)
    - Pocas veces (1)
    - De vez en cuando (2)
    - Constantemente (3)

3. Actualmente, ¿cuánto tiempo dedica a la lectura cada día?
    - Nada (0)
    - Menos de 30 minutos (1)
    - Entre 30 minutos y 2 horas (2)
    - Más de 2 horas (3)

4. ¿Cuánto tiempo dedica diariamente a leer en un dispositivo electrónico (teléfono, tablet, computador)?
    - Nada (0)
    - Menos de 30 minutos (1)
    - Entre 30 minutos y 2 horas (2)
    - Más de 2 horas (3)

5. ¿Cuánto tiempo dedica diariamente a leer en papel?
    - Nada (0)
    - Menos de 30 minutos (1)
    - Entre 30 minutos y 2 horas (2)
    - Más de 2 horas (3)

Fondecyt Fondo Nacional de Desarrollo Científico y Tecnológico
Universidad de Concepción
Universidad de Chile
UNIVERSIDAD DEL BÍO-BÍO

6. En general, ¿cuánto le gusta leer?
   - Nada (0)
   - Me gusta poco (1)
   - Ni mucho ni poco (2)
   - Me gusta mucho (3)

7. ¿Acostumbra a comentar con otras personas lo que usted ha leído?
   - Nunca (0)
   - Pocas veces (1)
   - Muchas veces (2)
   - Siempre (3)

8. ¿Cuántos libros tiene en casa?
   - Nada (0)
   - Pocos (1)
   - Varios (2)
   - Muchos (3)

9. ¿Qué es lo que usted principalmente lee?
   - Nada (0)
   - Sólo cuentas de luz, agua, boletas y correspondencia (1)
   - Titulares de diario, noticias y mensajes al teléfono (2)
   - Principalmente libros, novelas y otros (3)

10. ¿Consigue material de lectura (libros, revistas, etc.) en lugares como biblioteca, club de lectores, iglesia u otros?
   - Nunca (0)
   - Pocas veces (1)
   - Muchas veces (2)
   - Siempre (3)

II. Autopercepción de las dificultades de lectura

1. ¿Tiene dificultades para leer?
   - Nunca (0)
   - Rara vez (1)
   - Algunas veces (2)
   - Muchas veces (3)
   - Siempre (4)

2. ¿Le cuesta reconocer algunas letras cuando está leyendo?
    - Nunca (0)
    - Rara vez (1)
    - Algunas veces (2)
    - Muchas veces (3)
    - Siempre (4)

3. ¿Encuentra palabras desconocidas en los textos?
    - Nunca (0)
    - Rara vez (1)
    - Algunas veces (2)
    - Muchas veces (3)
    - Siempre (4)

4. ¿Le cuesta comprender frases largas?
    - Nunca (0)
    - Rara vez (1)
    - Algunas veces (2)
    - Muchas veces (3)
    - Siempre (4)

5. ¿Se distrae con facilidad cuando está leyendo?
    - Nunca (0)
    - Rara vez (1)
    - Algunas veces (2)
    - Muchas veces (3)
    - Siempre (4)

6. ¿Le cuesta recordar el contenido de lo que ha leído?
    - Nunca (0)
    - Rara vez (1)
    - Algunas veces (2)
    - Muchas veces (3)
    - Siempre (4)

7. Cuando el texto le resulta difícil, ¿abandona la lectura?
    - Nunca (0)
    - Rara vez (1)
    - Algunas veces (2)
    - Muchas veces (3)
    - Siempre (4)

Fondecyt
Fondo Nacional de Desarrollo Científico y Tecnológico

Universidad de Concepción

Universidad de Chile

Universidad del Bío-Bío
